# Antibody Arrays Identified Cycle-Dependent Plasma Biomarker Candidates of Peritoneal Endometriosis

**DOI:** 10.3390/jpm12060852

**Published:** 2022-05-24

**Authors:** Maja Pušić, Teja Klančič, Tamara Knific, Andrej Vogler, Ronny Schmidt, Christoph Schröder, Tea Lanišnik Rižner

**Affiliations:** 1 Faculty of Medicine, Institute of Biochemistry and Molecular Genetics, University of Ljubljana, 1000 Ljubljana, Slovenia; maja.pusic@mf.uni-lj.si (M.P.); teja.klancic@mf.uni-lj.si (T.K.); tamaraknific@gmail.com (T.K.); 2 Department of Obstetrics and Gynecology, University Medical Centre Ljubljana, 1000 Ljubljana, Slovenia; andrej.vogler@guest.arnes.si; 3 Sciomics GmbH, 69151 Heidelberg, Germany; ronny.schmidt@sciomics.de (R.S.); schroeder@sciomics.de (C.S.)

**Keywords:** peritoneal endometriosis, antibody arrays, biomarkers, integrins, proteomics, discovery, menstrual phase

## Abstract

Endometriosis is an estrogen-dependent inflammatory disease affecting women in their reproductive age. Due to non-specific symptoms, women with endometriosis are often misdiagnosed or are accurately diagnosed only after several years. Diagnosis of peritoneal endometriosis is especially challenging and relies only on laparoscopic surgery. To date, different molecules have been proposed as potential non-invasive biomarkers of endometriosis; however, none have been confirmed as clinically useful. Therefore, this study aimed to discover novel plasma biomarker candidates for peritoneal endometriosis using an antibody array platform. This study included patients with endometriosis-like symptoms characterized by the absence (controls) or presence of peritoneal endometriosis (cases) after laparoscopic surgery and histological evaluation. Patients were further divided into secretory and proliferative groups, according to the phase of their menstrual cycle. Their plasma samples were collected and analyzed on an antibody array platform targeting more than 1350 proteins with over 1820 antibodies. In the proliferative group, the analysis revealed three differential proteins between cases and controls: ITB3, ITA2B2, and ACVL-1. In the secretory group, none of the examined proteins reached the log-fold change (logFC) and significance thresholds simultaneously. The potential of the identified differential proteins as plasma biomarker candidates for peritoneal endometriosis should be evaluated on a larger cohort, and their role in endometriosis should be investigated in further studies.

## 1. Introduction

Endometriosis is a gynecological inflammatory disease that is highly prevalent among young women [1]. The disease is defined by the presence of ectopic endometrial tissue outside the uterine cavity [2] and can be divided into three distinct clinical types: peritoneal, ovarian, and deep infiltrating endometriosis [2,3]. Nevertheless, endometriotic lesions can also be found on the kidneys, bladder, lungs, and even the brain [4,5], and their localization affects the symptoms of endometriosis, which are non-specific and include infertility, dysmenorrhea, pelvic pain, and gastrointestinal problems [6]. Therefore, endometriosis is a heterogeneous disease, with different etiologies, presentations, and locations [7,8]. For example, peritoneal endometriosis comprises lesions that superficially involve the peritoneum [9].

The clinical signs are unspecific, and thus misdiagnoses are common because the symptoms overlap with other diseases such as irritable bowel syndrome and pelvic infection [10]. Currently, endometriosis cannot be determined by clinical examinations or blood tests alone [11]. For ovarian endometriosis, different preoperative imaging techniques, such as ultrasound, computed tomography, and magnetic resonance, are being explored as potential diagnostic tools [12]. However, these detection methods are not reliable for the most prevalent type—peritoneal endometriosis [12]. Currently, the gold standard for conclusive diagnosis is still exploratory laparoscopic surgery with histological examination [13]. However, this surgical procedure carries potential risks for the patients, and thus the development of a non-invasive test is needed [7]. Earlier diagnosis and consequently earlier treatment of endometriosis would increase fertility, decrease pain, prevent disease progression, and improve the quality of life of endometriosis patients [14,15].

Biomarkers for non-invasive diagnosis can be found and quantified from different biological samples (e.g., urine, serum, and plasma) using targeted and global non-targeted approaches (transcriptomics, metabolomics, and proteomics) [14,16]. To date, more than 50 studies have attempted to find non-invasive biomarkers of endometriosis, as described in recent reviews [1,14,17,18,19]. Specifically, glycoproteins, growth factors, microRNAs, long non-coding RNAs, and certain proteins involved in immune system regulation and angiogenesis were investigated as potential biomarkers [1]. In our previous studies, we aimed to identify novel biomarkers among different cytokines and metabolites in peripheral blood and peritoneal fluid samples using multiplex immunoassays and mass_spectrometry methods [20,21,22,23]. Unfortunately, not a single biomarker exhibited sufficient sensitivity and specificity in diagnosing endometriosis [1]. Decades of research using the hypothesis-based/driven approach did not provide biomarkers with appropriate diagnostic characteristics, and these studies revealed that only panels of biomarkers can reach sufficient sensitivity and specificity [14]. Global omics studies, either targeted or non-targeted, are thus needed to examine large number of biomolecules using the so-called hypothesis-generating approach. The aim of our current study was to identify a novel biomarker panel for peritoneal endometriosis using a targeted proteomics antibody array platform. To the best of our knowledge, this is the first study to use antibody arrays to identify biomarkers of peritoneal endometriosis.

## 2. Materials and Methods

### 2.1. Patient Enrollment and Study Design

**Figure 1:**
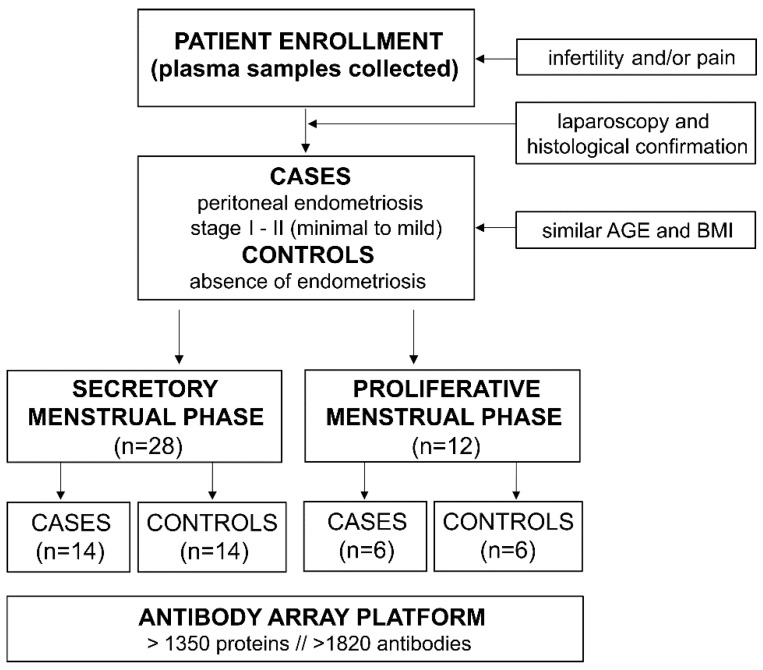
Flowchart of patient enrollment and study design.

Patient enrollment was conducted between 2016 and 2019 at University Medical Center Ljubljana. Only patients with endometriosis-like symptoms (infertility and/or chronic pelvic pain) were enrolled. All the patients underwent laparoscopic surgery, after which histological analysis revealed the presence of peritoneal endometriosis (cases) or absence of endometriosis (controls). There were no significant differences in age or body mass index (BMI) between cases and controls. Endometriosis patients were classified with stage I to II (minimal to mild) endometriosis according to the revised American Fertility Society score (rAFS). All the women included in the study were of Caucasian ethnicity and were not pregnant at the time of the surgery. The National Medical Ethics Committee of the Republic of Slovenia approved the study (no. 120-12772016-2), and all the participants signed their written informed consent before being enrolled in the study. The patients were divided according to the phase of their menstrual cycle into either the secretory (*n* = 28) or proliferative (*n* = 12) group, and their plasma samples were analyzed on an antibody array platform (Sciomics GmbH, Neckargemünd, Germany; Figure 1). The higher number of patients in the secretory menstrual phase led to a higher number of patients in the secretory group. The clinical characteristics of the patients included in the study are presented in Table 1.

### 2.2. Sample and Data Collection

One day to one week before surgery, blood samples were collected at the Department of Obstetrics and Gynecology at the University Medical Centre Ljubljana, Slovenia. For sample collection and overall processing, a strict standard operating procedure was followed as previously described [24,25]. Briefly, 4 mL of blood sample was obtained by venipuncture from the median cubital vein in BD vacutainer K2 EDTA tubes. Tubes were turned upside down 10 times for sufficient mixing with anticoagulant and immediately placed at 4 °C. All samples were processed within 1 h of collection. Obtained blood samples were centrifuged at 1400× *g* for 10 min at 4 °C. The plasma was collected, aliquoted into 100 µL volumes, and stored at −80 °C until further analysis. Only once frozen/thawed samples were used in this study. On the day of surgery, all the patients were asked to fill out a questionnaire regarding their general state of health, diet and lifestyle, stress level, medication, and different types of pain that are typical of endometriosis (i.e., frequency and intensity of dysmenorrhea, dyspareunia, dysuria, dyschezia, nausea/vomiting, and other types of lower abdominal pain). The appointed doctor filled out another questionnaire that provided other clinical and gynecological information, including age, BMI, age at menarche, regularity and length of the menstrual cycle, previous pregnancies or childbirth status, previous use of oral contraception and hormonal therapy, previous gynecological surgeries, and presence of additional pathologies.

### 2.3. Preparation of Samples for Antibody Microarray Analysis

In total, 50 µL of plasma samples was used for the analysis. Sample labeling and incubation were performed as previously described, with a few modifications [26]. Briefly, the protein concentration of plasma samples was determined using BCA assay. Samples were labeled at an adjusted protein concentration of 4 mg/mL with two dyes (scioDye 1 and scioDye 2) in a total volume of 65 µL for 2 h, after which the reaction was stopped with hydroxylamine. Excess dye was removed, and the buffer was exchanged to phosphate-buffered saline (PBS). Proliferative group samples were analyzed with a dual-color approach using a reference-based design on 12 scioDiscover antibody microarrays (Sciomics GmbH, Neckargemünd, Germany) targeting 1360 different proteins with 1830 antibodies. Secretory group samples were analyzed on 28 scioDiscover antibody microarrays targeting 1352 proteins with 1821 antibodies. Each antibody is represented on the array in four replicates. The arrays were blocked with scioBlock (Sciomics GmbH, Neckargemünd, Germany) on a Hybstation 4800 (Tecan, Grödig, Austria), and samples were incubated competitively using a dual-color approach for 3 h. Then, the slides were thoroughly washed with PBS containing Tween-20 and TritonX-100 (PBSTT), 0.1 × PBS, and water, and finally dried with nitrogen.

### 2.4. Data Acquisition and Statistical Analysis of Microarray Data

Slide scanning was conducted using a Powerscanner (Tecan, Grödig, Austria) with identical instrument laser power and adjusted photomultiplier tube (PMT) settings. Spot segmentation was performed with GenePix Pro 6.0 (Molecular Devices, Union City, CA, USA). Acquired raw data were analyzed using the linear models for microarray data (LIMMA) package of R-Bioconductor after uploading the median signal intensities. For normalization, a specialized invariant Lowess (locally weighted scatterplot smoothing) method was applied to the signal intensities as described before in detail [27]. For analysis of the samples, a one-factorial linear model was fitted with LIMMA, resulting in a two-sided *t*-test or F-test based on moderated statistics. All presented *p*-values were adjusted for multiple testing by controlling the false discovery rate (FDR) according to Benjamini and Hochberg. Differences in protein abundance between different sample groups are presented as log-fold changes (logFC) calculated to the base of 2. Proteins were defined as differential if logFC > 0.5 and the adjusted *p*-value < 0.05. Comparing cases versus controls, a logFC = 1 means that the case group has, on average, a 2^1^ = 2-fold higher signal than the control group. logFC = −1 represents 2^−1^ = ½ of the signal in the case as compared to the control group. The protein interaction network and gene ontology (GO) enrichment analysis between proteins reaching logFC > 0.5 and unadjusted *p*-values < 0.05 were performed using the STRING (Search Tool for the retrieval of Interacting Genes/Proteins) database version 11.5 (https://string-db.org/, accessed on 18 January 2022) [28].

The statistical analysis of microarray data may have yielded lower significance and logFC values because sample numbers included in the study were low and individual samples can introduce a large impact on the final results. Therefore, a study with a larger sample number should be performed to strengthen the data and confirm the results.

### 2.5. Statistical Analysis of Patients’ Clinical Data

Analysis of patients’ clinical data was performed as follows. First, the normality of distribution was checked with the Shapiro-Wilk test. Outliers were identified with the ROUT method and excluded from further analysis. The ROUT method based on the false discovery rate (FDR) was applied in order to enable the detection of one or more than one outliers. The ROUT coefficient Q was set at 1, aiming to show for no more than 1% of the identified outlier to be false. Then, either the unpaired *t*-test or Mann-Whitney test were used for the analysis of continuous variables of clinical data. Fisher’s exact test, the chi-squared test, or the chi-squared test for trend were used to compare categorical clinical variables. Statistical analysis was performed using GraphPad Prism 9 (GraphPad Software, San Diego, CA, USA). The level of significance was set at *p* < 0.05.

## 3. Results

### 3.1. Clinical Characteristics of Patients

Cases and controls from the proliferative and secretory groups in the discovery phase of the study were similar in terms of age and BMI. In the proliferative group, one case had been taking oral contraception and one control patient had been taking hormonal therapy in the last three months before surgery. In the secretory group, none of the patients had been taking oral contraception, whereas four cases and four controls (out of 28 patients) were taking hormonal therapy three months prior to surgery. In each group, one patient was classified with stage II endometriosis, whereas the rest of the patients had stage I endometriosis. In the proliferative group, none of the patients had previous pregnancies while in the secretory group two out of 14 controls had previously given birth. There were no significant differences between patients and controls in the frequency of oral and hormonal therapy use in the last three months before surgery, use of medications one week before surgery, sports or recreational activity, or smoking status.

### 3.2. Differentially Expressed Proteins Were Identified Only in the Proliferative Group of Patients

**Figure 2:**
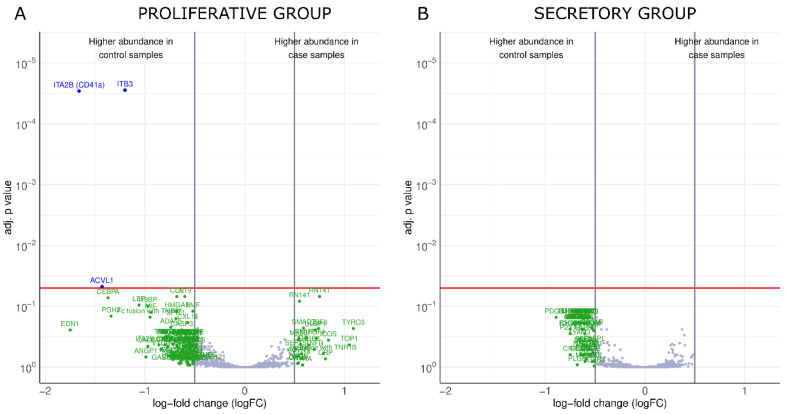
Proteins with distinct abundance variations between cases and controls. Volcano plots of the proliferative (**A**) and secretory (**B**) groups. The significance level of the *p*-value adjusted for multiple testing (adj. *p*-value) = 0.05 is indicated as a horizontal red line, and the log-fold change (logFC) cutoffs are indicated as vertical gray lines. Proteins with a positive logFC were more abundant in endometriosis patients, whereas proteins with a negative FC value were more abundant in control patients.

In the proliferative group, three proteins were more expressed in the plasma samples of controls compared to those of endometriosis patients: integrin beta 3 (ITB3), serine threonine-protein kinase receptor R3/activin receptor-like kinase 1 (ACVL-1/ALK-1), and integrin alpha-IIb (ITA2B) (Table 2). The results of the statistical analysis are summarized in the volcano plot (Figure 2A). In the secretory group, none of the proteins reached the logFC and adjusted *p*-value threshold simultaneously to be considered differentially expressed between cases and controls (Figure 2B). However, the secretory group had 52 proteins that reached logFC > 0.5 and exhibited a significant difference between cases and controls on the basis of unadjusted *p*-values < 0.05; the proliferative group had 75 such proteins (Table S1, Supplementary Materials).

### 3.3. The Levels of Identified Proteins Allowed for the Separation of Endometriosis Patients from Control Patients

**Figure 3:**
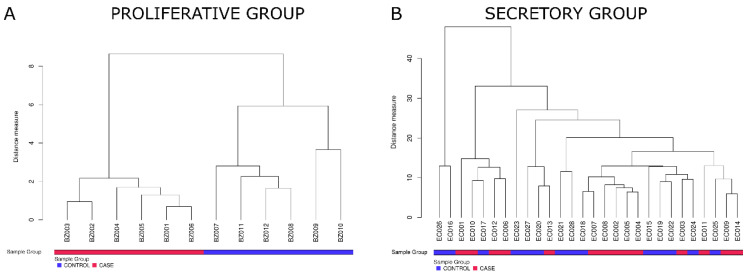
Hierarchical clustering based on differential proteins. (**A**) In the proliferative group, clustering was conducted on the basis of the expression of three differential proteins (listed in Table 2) that reached logFC > 0.5 and adjusted *p*-values < 0.05 between cases and controls. (**B**) In the secretory group, clustering was conducted on the basis of 52 proteins (listed in Table S1) that reached logFC > 0.5 and unadjusted *p*-values < 0.05.

In the proliferative group, hierarchical cluster analysis of the complete array data showed heterogeneous clustering (data not shown), whereas analysis based on the three differential proteins that reached adjusted *p*-values < 0.05 (listed in Table 2) revealed clustering of samples according to their disease status with a clear differentiation between control and endometriosis patients (Figure 3A). In the secretory group, hierarchical cluster analysis of the complete protein data set revealed heterogeneous clustering (data not shown). Because no proteins simultaneously reached logFC > 0.5 and adjusted *p*-values < 0.05 in the secretory group, additional cluster analysis was performed on proteins that reached logFC > 0.5 and unadjusted *p*-values < 0.05 (listed in Table S1, Supplementary Materials). The analysis showed samples with outlier behavior: EC005 and EC016. Other samples were distributed in five subclusters, implying stronger differences between patients than between cases and controls (Figure 3B).

**Figure 4:**
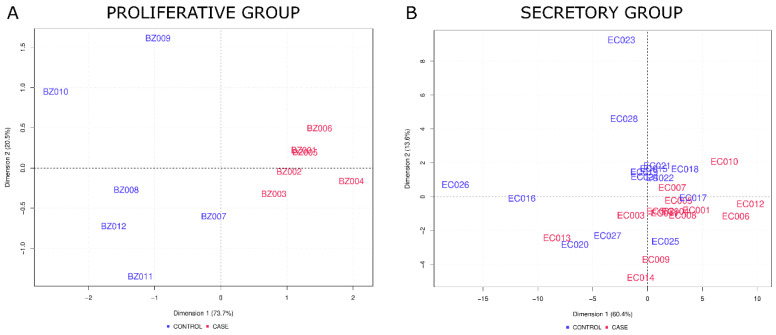
Principal component analysis based on differential proteins. (**A**) For the proliferative group, PCA analysis was performed on the basis of three differentially abundant proteins that reached logFC > 0.5 and adjusted *p*-values < 0.05 between cases and controls. (**B**) For the secretory group, PCA was performed on the basis of differential proteins that reached logFC > 0.5 and unadjusted *p*-values < 0.05. The percentages provided in the axis labels describe the ratio of total variance according to the respective principal component. Samples labeled with EC and BZ belong to the secretory and proliferative group, respectively. Controls and cases are indicated in blue and red, respectively.

Additionally, a principal component analysis (PCA) was performed for the complete array data for the proliferative and secretory groups, showing no clear distinction between cases and controls (data not shown). In the proliferative group, PCA based on three differentially abundant proteins that reached logFC > 0.5 and adjusted *p*-values < 0.05 clearly distinguished between cases and controls (Figure 4A). In the secretory group, PCA based on differential proteins that reached logFC > 0.5 and only unadjusted *p*-values < 0.05 showed a rough distinction between cases and controls. However, 4 out of 14 control samples clustered together with cases (Figure 4B).

In the secretory group, none of the proteins reached adjusted *p*-values < 0.05, and PCA did not clearly distinguish between cases and controls. Thus, we assessed whether any patient characteristics affect sample clustering. To achieve this, an additional PCA based on complete array data and different patient characteristics was performed (Figure S1, Supplementary Materials). The following patient data were used for the analysis: gynecological characteristics (age, use of oral contraception and hormonal therapy 3 months beforesurgery, use of medication 1 week before surgery, length of menstrual cycle, and duration of menstrual period), diet and lifestyle characteristics (coffee and tea consumption 1 day before surgery, alcohol consumption, use of soybean food supplements, smoking status, frequency of sport/recreation), and different stress parameters (sense of (not) being in control, unable to cope with problems, and feeling whether life is going in the right direction). None of the mentioned characteristics produced a clear clustering of samples, suggesting that no single individual patient characteristic introduced the identified large differences between samples.

### 3.4. Studying Protein Interactions Revealed That the Differential Proteins Play Roles in Inflammation, the Immune System, Cell Adhesion, Platelet Aggregation, and Angiogenesis

**Figure 5:**
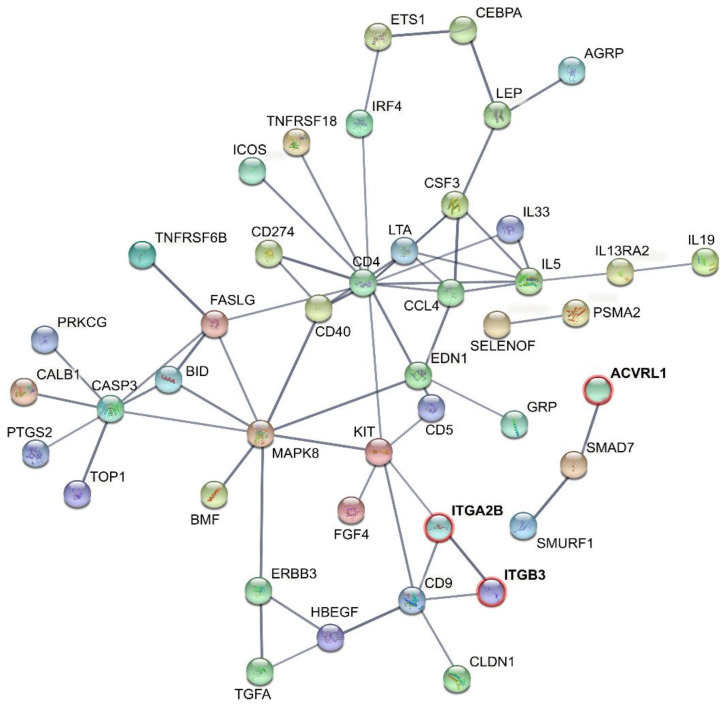
Interactions of the identified differential proteins between patients with peritoneal endometriosis and controls patients in the proliferative group. The network includes proteins that reached a significant difference between cases and controls according to adjusted *p* < 0.05 and unadjusted *p* < 0.05. The confidence score threshold was set at 0.7 (high). The figure was acquired at http://string-db.org*/,* accessed on 18 January 2022 [28].

Predicted protein interactions (PPI) between cases and controls for the proliferative group included differential proteins that reached logFC > 0.5 and both adjusted and unadjusted *p* < 0.05 (Figure 5). The proliferative group protein network showed a clustering coefficient of 0.529 containing 76 nodes and 226 edges (versus 66 expected edges) with PPI enrichment *p* < 1.0 × 10^−16^. Proteins that simultaneously reached logFC > 0.5 and adjusted *p* < 0.05 (ITB3, ITA2B, and ACVL-1) are known to be connected to cell adhesion, platelet aggregation, angiogenesis, and wound healing. PPI revealed connection between the three significantly different proteins and those that only reached unadjusted *p* < 0.05 (Figure 5). The STRING network for the proliferative group showed an enrichment in proteins involved in biological processes such as responses to cytokines and molecular functions connected to receptor and cytokine binding (Table 3).

Additionally, STRING analysis of proteins that reached unadjusted *p* < 0.05 in the secretory group revealed an average local clustering coefficient of 0.432 containing 51 nodes and 113 edges (versus 25 expected edges) with PPI enrichment *p* < 1.0 × 10^−16^ (Figure S2, Supplementary Materials). These PPI values indicate a high number of protein interactions and at least partial biological connection among proteins in both groups. In the secretory group, the network is significantly enriched with proteins involved in biological processes connected to inflammation and immune system processes and molecular functions connected to cytokine activity and binding (Table 3).

## 4. Discussion

Earlier diagnosis and adequate treatment of endometriosis could be achieved with the development of non-invasive diagnostic tests based on a panel of reliable biomarkers [29]. This would consequently improve patient quality of life and lower the direct and indirect costs associated with the management of endometriosis [30]. In the last 30 years, different molecules have been discovered and evaluated as potential non-invasive biomarkers for endometriosis; however, none of them have been validated for clinical use [1,14,16,18,19].

In this study, we aimed to identify a panel of plasma biomarkers for peritoneal endometriosis using an antibody array platform. We used a hypothesis-generating approach and examined levels of more than 1350 proteins in plasma samples of patients with and without confirmed peritoneal endometriosis. With this approach, we have included proteins that have not yet been reported as relevant in the pathophysiology of endometriosis. Previously, another research group tried to identify novel biomarkers of endometriosis using L-series-1000 and Quantibody 660 antibody array platforms (RayBiotech, Norcross, GA, USA), which target 1000 and 660 proteins, respectively [31]. Their study included 68 endometriosis patients with ASRM stage I-II (*n* = 31) and stage III-IV (*n* = 37) and 35 control patients with endometriosis-like symptoms. The data acquired with the L-series-1000 platform were found to be unreliable, whereas the Quantibody 660 platform discovered 309 differential proteins between the case and control groups. Validation was performed for 10 proteins, and only IL-31 was confirmed as a possibly useful biomarker for endometriosis. The authors of the study reported problems with the reproducibility of data acquired by the antibody array platforms and problems with validation using custom-made multiplex and ELISA immunoassays. They suggested using different antibody arrays for biomarker discovery in plasma samples of endometriosis patients, and this was performed in our study.

The antibody array platform (scioDiscover platform, Sciomics GmbH, Neckargemünd, Germany) used in our study covers a unique set of proteins, including secreted proteins, receptors, cell surface markers, and intracellular signaling pathway molecules. This antibody array platform runs four technical replicates for each antibody in the same assay. The coefficient of variation of the replicates is on average below 10% (Figure S3, Supplementary Materials). It has a very high sensitivity, especially for detecting proteins in plasma and serum samples. So far, a number of studies have used this platform to discover novel pathways or biomarker signatures specific for certain diseases [32,33,34]. Additionally, we have previously used the same platform to successfully identify biomarker candidates for endometriosis in peritoneal fluid samples [26].

Furthermore, our study aimed to discover potential biomarkers of early_stage (minimal to mild) peritoneal endometriosis. Currently, there are no clinically useful non-invasive biomarkers that could reveal early_stage endometriosis [35]. Women with superficial peritoneal endometriosis are often misdiagnosed due to inadequate intraoperative descriptions of endometrial lesions provided by surgeons or false attributions of non-pigmented lesions as inactive [36]. Even though transvaginal ultrasound can be very useful in diagnosing ovarian and deep infiltrating endometriosis, it is not reliable for detecting peritoneal endometriosis [37]. The positive predictive value of visual inspection by laparoscopy is higher in patients with advanced stages of endometriosis, whereas multiple biopsies of suggestive lesions are sometimes necessary in patients with minimal to mild endometriosis before the lesions are properly diagnosed [38,39]. In addition, the majority of adolescents have stage I–II endometriosis that stays undiscovered for years and causes subsequent infertility [40]. Therefore, it is important to develop non-invasive diagnostic tests for minimal to mild peritoneal endometriosis that would enable earlier diagnosis and prevent progression of the disease to more severe stages [41].

To ensure the validity of our results and minimize pre-analytical bias, we collected peripheral blood samples according to the established and validated strict standard operating procedure. Although serum is still one of the most analyzed biological materials, problems arise due to its inherently variable composition that depends on blood-clotting [42]. Therefore, we used plasma samples in our study.

Another strength of this study are the homogenous and very well-defined case and control groups. For well-designed case-control studies, it is recommended that controls and cases are selected from the same population [43]. In this study, both groups of patients (case and control) had endometriosis-like symptoms (infertility and/or pain) and a similar age and BMI. Furthermore, we stratified patients according to their menstrual cycle phase into secretory and proliferative groups. Differential proteins were found only in the proliferative group, which indicates that the discovered biomarker candidates are cycle_dependent. There are two reasons why it is important to consider possible cycle_dependence in the discovery of endometriosis biomarkers. (1) Endometriosis is an estrogen-dependent disease, and previous studies confirmed that the secretion and synthesis of endometrial proteins changes during the menstrual cycle [44]. (2) Specific molecular patterns (at transcriptomic, epigenomic, and proteomic levels) in the eutopic endometrium of endometriosis patients compared to normal endometrium of healthy women are associated with the different phases of the menstrual cycle [45].

The weakness of this study is the use of a different number of plasma samples from the secretory and proliferative groups for the discovery phase. Although laparoscopy can be done in any phase of the menstrual cycle [46], we have enrolled a lower number of patients in the proliferative phase, which resulted in a lower number of suitable patients for the discovery phase. Another weakness is that the discovered differential proteins were not validated. For successful validation, reliable ELISA immunoassays without lot-to-lot variability and with high intra- and inter-assay reproducibility must be available. Additionally, the reliability of the immunoassays is predicated on antibodies that must have high affinity towards an analyte [47]. The market is overflowing with ELISAs of questionable quality and antibodies that did not pass the established quality standards [48]. Another problem can be the sensitivity of available assays for validation when very sensitive discovery assays, such as the scioDiscover platform, are used.

In the proliferative group in this study, we identified only three proteins that were differentially abundant, i.e., exhibited lower levels in endometriosis patients compared to controls: ITB3, ACVL-1, and ITA2B. Integrins are transmembrane glycoproteins composed of non-covalently bound α and β subunits. These cell adhesion molecules are involved in cell proliferation, differentiation, migration, and cell survival [49]. Integrin β3 (ITB3) and integrin αIIb (ITA2B) are found on the surface of platelets and endothelial cells and play a role in angiogenesis and platelet aggregation [50,51]. Previous studies demonstrated the presence of platelet aggregations at endometriotic lesions and the involvement of platelets in the development of endometriosis [52]. Although the identified integrins were not previously found in the peripheral blood or peritoneal fluid of endometriosis patients, their involvement in platelet aggregation makes them interesting potential biomarkers of endometriosis. Interestingly, Lessey et al. documented the absence of integrin αvβ3 in endometrial tissue of women with minimal to mild endometriosis and suggested endometrial integrin αvβ3 as a non-invasive marker for early_stage endometriosis [53]. Our study is the first to report decreased levels of β3 and αIIb subunits in the plasma samples of patients with peritoneal endometriosis and in their proliferative menstrual phase.

Additionally, we are the first to report decreased levels of ACVL-1 in plasma samples of endometriosis patients. ACVL-1 is a type I receptor of the transforming growth factor-β family of proteins and has not yet been associated with endometriosis. It is mainly expressed in endothelial cells but can be found in other types of cells, such as smooth muscle cells, monocytes, myoblasts, skin fibroblasts, and macrophages [54]. ACVL-1 is a cell surface receptor that has an important role in angiogenesis, wound healing, and tumor growth [55].

All three identified differential proteins from the proliferative group are cell surface proteins. Although a few ELISA assays have been developed for the detection of membrane-bound proteins, each assay must be adapted for the protein of interest. Additionally, methods of protein solubilization can impact the accessibility for antibody binding and result in assays with low detection ranges [56]. The three differentially abundant proteins were detected in plasma samples in the antibody array analysis; however, the lack of a reliable commercial ELISA assay for their detection precluded further validation of ITB3, ITA2B, and ACVL-1 in a larger cohort of patients.

In both the proliferative and secretory groups, proteins that differed between cases and controls based on unadjusted *p* < 0.05 and logFC < 0.5 had high numbers of PPI in the obtained STRING networks. The acquired data provide a wealth of information considering the pathophysiology of peritoneal endometriosis. Protein networks of both groups comprised large numbers of cytokines, chemokines, and proteins related to the immune system. The role of cytokines and chemokines in the pathogenesis of endometriosis has already been described [57,58], and several molecules have been proposed as possible markers of endometriosis [1,59,60]. However, as cytokines are mediators of inflammatory and immune responses and can reflect the presence of various inflammatory and autoimmune diseases, it is difficult to identify cytokines that are only associated with endometriosis [18]. Peritoneal fluid is considered a better source for cytokine detection than peripheral blood because immune cells of peritoneal fluid specifically reflect the pathology of endometriosis [57]. In our previous study, peritoneal fluid was proven to be more appropriate for the discovery of novel biomarkers of endometriosis using antibody array platforms [26]. We were able to identify 16 differential proteins between control and endometriosis patients and successfully validated three biomarker candidates in a larger cohort. Since peritoneal fluid much better reflects the inflammatory environment associated with endometriosis, molecules found to be differentially expressed in peritoneal fluid warrant further investigation [61]. The importance of these molecules should be investigated in blood samples, as they can play a role in the pathophysiology of endometriosis or be used as potential biomarkers for the non-invasive diagnosis of endometriosis.

In conclusion, we aimed to discover plasma biomarker candidates of peritoneal endometriosis using scioDiscover, an antibody array platform. We identified three differentially abundant proteins (ITB3, ITA2B, and ACVL-1) between control and peritoneal endometriosis patients in their proliferative menstrual phase. The abundance of these identified proteins did not significantly differ between cases and controls in the secretory group, implying cycle_dependence. The discovered differential proteins are known to be associated with different processes involved in the pathophysiology of endometriosis, e.g., cell adhesion, platelet aggregation, and angiogenesis. Validation of the discovered differential proteins on a larger cohort of patients was not performed due to a lack of suitable ELISA assays; however, the connection of these proteins with endometriosis should be further explored.

## Figures and Tables

**Table 1:** Clinical characteristics of patients included in the study.

Characteristics	Unit	Menstrual Phase	Subgroup	Patients with Peritoneal Endometriosis	Controls	*p*-Value
Total number of patients	n	Secretory	-	14	14	-
		Proliferative	-	6	6	-
Age (mean ± SD)	years	Secretory	-	31.93 ± 4.10	30.14 ± 4.15	Mann-Whitney test (*p* = 0.2114)
		Proliferative	-	30 ± 3.46	28 ± 2.19	unpaired *t*-test (*p* = 0.2596)
BMI (mean ± SD)	kg/m^2^	Secretory	-	22.55 ± 2.39	22.31 ± 2.72	unpaired *t*-test (*p* = 0.8062)
		Proliferative	-	21.27 ± 1.02	22.35 ± 2.42	unpaired *t*-test (*p* = 0.3389)
rAFS score	n (%)	Secretory	I	13 (92.86)	0 (0)	-
			II	1 (7.14)	0 (0)	-
		Proliferative	I	5 (83.30)	0 (0)	-
			II	1 (16.70)	0 (0)	-
Oral contraception 3 months before surgery	n (%)	Secretory	Yes	0 (0)	0 (0)	Fisher’s exact test (*p* > 0.9999)
			No	14 (100)	14 (100)	
		Proliferative	Yes	1 (16.70)	0 (0)	Fisher’s exact test (*p* > 0.9999)
			No	5 (83.30)	6 (100)	
Hormonal therapy 3 months before surgery	n (%)	Secretory	Yes	4 (28.57)	4 (28.57)	Fisher’s exact test (*p* > 0.9999)
			No	10 (71.43)	10 (71.43)	
		Proliferative	Yes	0 (0)	1 (16.70)	Fisher’s exact test (*p* > 0.9999)
			No	6 (100)	5 (83.30)	
Medication 1 week before surgery	n (%)	Secretory	Yes	4 (28.57)	4 (28.57)	Fisher’s exact test (*p* > 0.9999)
			No	10 (71.43)	10 (71.43)	
		Proliferative	Yes	4 (66.7)	2 (33.30)	Fisher’s exact test (*p* = 0.5671)
			No	2 (33.30)	4 (66.70)	
Smoking status	n (%)	Secretory	Non-smoker	7 (50)	5 (35.71)	Chi-squared test for trend (*p* = 0.1222)
			Smoker	4 (28.57)	4 (28.57)	
			Occasionally (weekly)	3 (21.43)	1 (7.14)	
			Occasionally (monthly)	0 (0)	2 (14.29)	
			Former smoker	0 (0)	2 (14.29)	
	n (%)	Proliferative	Non-smoker	4 (66.70)	3 (50)	Chi-squared test for trend (*p* = 0.5582)
			Smoker	2 (33.30)	3 (50)	
			Occasionally (weekly)	0 (0)	0 (0)	
			Occasionally (monthly)	0 (0)	0 (0)	
			Former smoker	0 (0)	0 (0)	
Sport/recreation	n (%)	Secretory	Regularly	8 (57.14)	9 (64.29)	Chi-squared test for trend (*p* = 0.5016)
			Occasionally	5 (35.71)	5 (35.71)	
			No	1 (7.15)	0 (0)	
	n (%)	Proliferative	Regularly	3 (50)	1 (16.70)	Chi-squared test for trend (*p* = 0.0929)
			Occasionally	3 (50)	3 (50)	
			No	0 (0)	2 (33.30)	

**Abbreviations:** n, number; SD, standard deviation; rAFS, revised American Fertility Society.

**Table 2:** Proteins with differential abundance between cases and controls in the proliferative group.

Protein Abbreviation (Uniprot)	Full Protein Name	Uniprot ID	logFC	FC	P
ITB3	Integrin beta-3	P05106	−1.20	0.44	2.8 × 10^−5^
ACVL-1	Serine threonine-protein kinase receptor R3	P37023	−1.43	0.37	4.7 × 10^−2^
ITA2B	Integrin alpha-IIb	P08514	−1.66	0.32	2.9 × 10^−5^

**Abbreviations:** FC, fold change calculated from logFC; logFC, logarithmic fold change calculated to the base of 2; P, *p*-values adjusted for multiple testing.

**Table 3:** Functional enrichments found in protein networks of the proliferative and secretory and groups.

**BIOLOGICAL PROCESSES**		
**PROLIFERATIVE GROUP**		
**Gene ontology** **(GO) term**	**Description**	**Protein count in the network**
GO:0034097	Response to cytokine	34 of 1101
GO:0007166	Cell surface receptor signaling pathway	45 of 2325
GO:0050896	Response to stimulus	70 of 8046
GO:0071345	Cellular response to cytokine stimulus	31 of 1013
GO:0051716	Cellular response to stimulus	64 of 6489
**SECRETORY GROUP**		
**Gene ontology** **(GO) term**	**Description**	**Protein count in the network**
GO:0006950	Response to stress	31 of 3485
GO:0050900	Leukocyte migration	12 of 316
GO:0002376	Immune system process	26 of 2481
GO:0071345	Cellular response to cytokine stimulus	17 of 1013
GO:0019221	Cytokine-mediated signaling pathway	14 of 678
**MOLECULAR FUNCTIONS**		
**PROLIFERATIVE GROUP**		
**Gene ontology** **(GO) term**	**Description**	**Protein count in the network**
GO:0005102	Signaling receptor binding	37 of 1581
GO:0030545	Receptor regulator activity	23 of 536
GO:0048018	Receptor ligand activity	22 of 490
GO:0005126	Cytokine receptor binding	15 of 264
GO:0005515	Protein binding	58 of 7026
**SECRETORY GROUP**		
**Gene ontology** **(GO) term**	**Description**	**Protein count in the network**
GO:0005125	Cytokine activity	8 of 233
GO:0048018	Receptor ligand activity	10 of 490
GO:0005102	Signaling receptor binding	16 of 1581
GO:0005126	Cytokine receptor binding	7 of 264
GO:0005515	Protein binding	34 of 7026

## Data Availability

The data are presented within the manuscript. The raw datasets used for this study are available upon request from the corresponding author.

## Supplementary Materials

The following supporting information can be downloaded at: https://www.mdpi.com/article/10.3390/jpm12060852/s1. Table S1: Differential proteins in the secretory and proliferative phases (unadjusted *p* values); Figure S1: PCA analysis based on complete array data and different patient characteristics; Figure S2: Interactions of the identified differential proteins between patients with peritoneal endometriosis and controls patients in the secretory group; Figure S3: Array-specific distributions of coefficients of variation (CV) for the four technical replicates measured by each antibody.
