# Supplementary material for: Antibody Arrays Identified Cycle-Dependent Plasma Biomarker Candidates of Peritoneal Endometriosis
Source: J Pers Med. 2022 May 24;12(6):852. doi: 10.3390/jpm12060852 (PMC9225192; doi:10.3390/jpm12060852)
Supplement: Supplementary file 1 [file jpm-12-00852-s001.zip › jpm-1716952-supplementary.pdf]

Supplementary Materials

Table S1. Differential proteins in the secretory and proliferative phase (unadjusted p-values)

SECRETORY MENSTRUAL PHASE						
**Protein abbreviation**	**Protein Full Name**	**Uniprot ID**	**logFC**	**FC**	**p-value**	**adj.p-val**
**PDCD1**	Programmed cell death protein 1	Q15116	-0.89	0.539614	0.00099	0.15
**BDNF**	Brain-derived neurotrophic factor	P23560	-0.78	0.582367	0.00054	0.15
**P2Y12**	P2Y purinoceptor 12	Q9H244	-0.75	0.594604	0.0063	0.28
**B3GA1**	Galactosylgalactosylxylosylprotein 3-beta-glucuronosyltransferase 1	Q9P2W7	-0.75	0.594604	0.0047	0.24
**CRP**	C-reactive protein	P02741	-0.75	0.594604	0.028	0.62
**CH3L1**	Chitinase-3-like protein 1	P36222	-0.75	0.594604	0.0042	0.24
**MELPH**	Melanophilin, MLPH	Q9BV36	-0.72	0.607097	0.00061	0.15
**CD33**	Myeloid cell surface antigen CD33	P20138	-0.71	0.61132	0.0027	0.19
**CRLF2**	Cytokine receptor-like factor 2	Q9HC73	-0.7	0.615572	0.0028	0.19
**MLP3B**	Microtubule-associated proteins 1A/1B light chain 3B	Q9GZQ8	-0.7	0.615572	0.0023	0.19
**GLPA**	Glycophorin-A	P02724	-0.69	0.619854	0.0019	0.19
**PLGF**	Placenta growth factor	P49763	-0.68	0.624165	0.16	0.92
**CEAM5**	Carcinoembryonic antigen-related cell adhesion molecule 5	P06731	-0.67	0.628507	0.0017	0.19
**PYRG1**	CTP synthase 1	P17812	-0.66	0.632878	0.0026	0.19
**RBM3**	RNA-binding protein 3	P98179	-0.65	0.63728	0.0019	0.19
**PTEN**	Phosphatidylinositol 3,4,5-trisphosphate 3-phosphatase and dual-specificity protein phosphatase PTEN	P60484	-0.65	0.63728	0.0021	0.19
**TSN16**	Tetraspanin-16	Q9UKR8	-0.65	0.63728	0.0013	0.15
**LYAM3**	P-selectin	P16109	-0.65	0.63728	0.008	0.34
**IL18**	Interleukin-18	Q14116	-0.65	0.63728	0.00074	0.15
**IFIT2**	Interferon-induced protein with tetratricopeptide repeats 2	P09913	-0.65	0.63728	0.003	0.19
**RRAGC**	Ras-related GTP-binding protein C	Q9HB90	-0.64	0.641713	0.00044	0.15
**DHRS2**	Dehydrogenase/reductase SDR family member 2, mitochondrial	Q13268	-0.64	0.641713	0.00023	0.15
**MMP1**	Interstitial collagenase	P03956	-0.64	0.641713	0.0004	0.15
**CEAM1**	Carcinoembryonic antigen-related cell adhesion molecule 1	P13688	-0.63	0.646176	0.027	0.62
**TNFB**	Lymphotoxin-alpha	P01374	-0.63	0.646176	0.0041	0.24
**SPA9**	Serpin A9	Q86WD7	-0.63	0.646176	0.015	0.45
**TMM54**	Transmembrane protein 54	Q969K7	-0.62	0.650671	0.0002	0.15
**ID3**	DNA-binding protein inhibitor ID-3	Q02535	-0.62	0.650671	0.0028	0.19
**MAD4**	Max dimerization protein 4	Q14582	-0.62	0.650671	0.0024	0.19
**PRTN3**	Myeloblastin	P24158	-0.62	0.650671	0.0049	0.24
**IL10**	Interleukin-10	P22301	-0.61	0.655197	0.028	0.62
**MK12**	Mitogen-activated protein kinase 12	P53778	-0.61	0.655197	0.00078	0.15
**NFAC4**	Nuclear factor of activated T-cells, cytoplasmic 4	Q14934	-0.61	0.655197	0.019	0.52
**IFNA1**	Interferon alpha-1/13	P01562	-0.61	0.655197	0.0013	0.15

**SDC1**	Syndecan-1	P18827	-0.61	0.655197	0.013	0.44
**CCL7**	C-C motif chemokine 7	P80098	-0.61	0.655197	0.026	0.62
**FABPI**	Fatty acid-binding protein, intestinal	P12104	-0.61	0.655197	0.0062	0.28
**AKA12**	A-kinase anchor protein 12	Q02952	-0.6	0.659754	0.003	0.19
**CCL11**	Eotaxin	P51671	-0.59	0.664343	0.00084	0.15
**KI67**	Proliferation marker protein Ki-67	P46013	-0.59	0.664343	0.04	0.75
**GAS6**	Growth arrest-specific protein 6	Q14393	-0.59	0.664343	0.015	0.45
**MRPP3**	Mitochondrial ribonuclease P catalytic subunit	O15091	-0.58	0.668964	0.019	0.52
**AREG**	Amphiregulin	P15514	-0.57	0.673617	0.00064	0.15
**MICA**	MHC class I polypeptide-related sequence A	Q29983	-0.57	0.673617	0.028	0.62
**TR11B**	Tumor necrosis factor receptor superfamily member 11B	O00300	-0.55	0.68302	0.028	0.62
**CD15**	/	/	-0.55	0.68302	0.017	0.5
**K2C8**	Keratin, type II cytoskeletal 8	P05787	-0.55	0.68302	0.0012	0.15
**ULBP1**	UL16-binding protein 1	Q9BZM6	-0.53	0.692555	0.018	0.52
**C2C4B**	C2 calcium-dependent domain-containing protein 4B	A6NLJ0	-0.53	0.692555	0.0038	0.23
**CD28**	T-cell-specific surface glycoprotein CD28	P10747	-0.52	0.697372	0.0044	0.24
**DAF**	Complement decay-accelerating factor, CD55	P08174	-0.51	0.700672	0.00540	0.25223
**FABPL**	Fatty acid-binding protein, liver	P07148	-0.51	0.702222	0.012	0.43
**PROLIFERATIVE MENSTRUAL PHASE**						
**Protein abbreviation**	**Protein Full Name**	**Uniprot ID**	**logFC**	**FC**	**p-value**	**adj.p-val**
**TYRO3**	Tyrosine-protein kinase receptor	Q06418	1.09	2.25	0.002864	0.231156
**TOP1**	DNA topoisomerase 1	P11387	1.05	2.18	0.016278	0.429842
**ICOS**	Inducible T-cell costimulator	Q9Y6W8	0.84	1.86	0.010292	0.360623
**RN141**	RING finger protein 141	Q8WVD5	0.75	1.74	0.000212	0.069074
**CSF3**	Granulocyte colony-stimulating factor	P09919	0.74	1.73	0.002918	0.231156
**FGF4**	Fibroblast growth factor 4	P08620	0.72	1.71	0.008054	0.33349
**ULBP1**	UL16-binding protein 1	Q9BZM6	0.71	1.69	0.003365	0.245555
**TNFB**	Lymphotoxin-alpha	P01374	0.70	1.68	0.032361	0.510999
**ERBB3**	Receptor tyrosine-protein kinase erbB-3	P21860	0.64	1.61	0.015696	0.429842
**SMUF1**	E3 ubiquitin-protein ligase SMURF1	Q9HCE7	0.62	1.58	0.005909	0.329705
**SMAD7**	Mothers against decapentaplegic homolog 7	O15105	0.59	1.55	0.002633	0.228419
**ASNS**	Asparagine synthetase [glutamine-hydrolyzing]	P08243	0.57	1.53	0.046593	0.605458
**CD4**	T-cell surface glycoprotein CD4	P01730	0.56	1.52	0.014497	0.419263
**RN141**	RING finger protein 141	Q8WVD5	0.55	1.50	0.000361	0.082273
**MK08**	Mitogen-activated protein kinase 8	P45983	0.54	1.49	0.009216	0.344338
**SEP15**	Selenoprotein F	O60613	0.51	1.46	0.029879	0.504069
**BLNK**	B-cell linker protein	Q8WV28	-0.51	0.68	0.005169	0.329705
**MMP1**	Interstitial collagenase	P03956	-0.51	0.68	0.018353	0.445853
**DAND5**	DAN domain family member 5	Q8N907	-0.51	0.68	0.022762	0.455168

**BID**	BH3-interacting domain death agonist	P55957	-0.51	0.68	0.024211	0.455168
**BMF**	Bcl-2-modifying factor	Q96LC9	-0.52	0.68	0.000795	0.120637
**IL25**	Interleukin-25	Q9H293	-0.52	0.68	0.005581	0.329705
**FGF23**	Fibroblast growth factor 23	Q9GZV9	-0.53	0.67	0.037835	0.555935
**IL5**	Interleukin-5	P05113	-0.53	0.67	0.040623	0.566374
**CD5**	T-cell surface glycoprotein CD5	P06127	-0.53	0.67	0.04419	0.587694
**IRF4**	Interferon regulatory factor 4	Q15306	-0.54	0.67	0.006334	0.329705
**CEAM7**	Carcinoembryonic antigen-related cell adhesion molecule 7	Q14002	-0.54	0.67	0.009578	0.349011
**BGLR**	Beta-glucuronidase	P08236	-0.54	0.67	0.022315	0.455168
**1433B**	14-3-3 protein beta/alpha	P31946	-0.55	0.66	0.012148	0.390734
**KPCG**	Protein kinase C gamma type	P05129	-0.55	0.66	0.016945	0.434598
**PSA2**	Proteasome subunit alpha type-2	P25787	-0.55	0.66	0.032815	0.510999
**CD53**	Leukocyte surface antigen CD53	P19397	-0.56	0.66	0.006555	0.33099
**GRP**	Gastrin-releasing peptide	P07492	-0.56	0.66	0.011694	0.387404
**TNR5**	Tumor necrosis factor receptor superfamily member 5	P25942	-0.56	0.66	0.018041	0.444205
**CXL16**	C-X-C motif chemokine 16	Q9H2A7	-0.57	0.66	0.001841	0.186346
**PGK1**	Phosphoglycerate kinase 1	P00558	-0.58	0.65	0.016798	0.434598
**IL17C**	Interleukin-17C	Q9P0M4	-0.59	0.65	0.008499	0.344097
**ICAM3**	Intercellular adhesion molecule 3	P32942	-0.59	0.65	0.00926	0.344338
**ETS1**	Protein C-ets-1	P14921	-0.59	0.65	0.025355	0.461959
**IL19**	Interleukin-19	Q9UHD0	-0.60	0.64	0.000175	0.069074
**HBEGF**	Proheparin-binding EGF-like growth factor	Q99075	-0.60	0.64	0.006903	0.33099
**OX2G**	OX-2 membrane glycoprotein	P41217	-0.60	0.64	0.009035	0.344338
**CRTAM**	Cytotoxic and regulatory T-cell molecule	O95727	-0.60	0.64	0.039634	0.566374
**I13R2**	Interleukin-13 receptor subunit alpha-2	Q14627	-0.61	0.64	0.021524	0.455168
**TGFA**	Protransforming growth factor alpha	P01135	-0.61	0.64	0.023683	0.455168
**IL33_MOUSE**	Interleukin-33	Q8BVZ5	-0.62	0.63	0.007876	0.33349
**CCL4**	C-C motif chemokine 4	P13236	-0.62	0.63	0.031859	0.510999
**CASP3**	Caspase-3	P42574	-0.63	0.63	0.003504	0.245555
**IL1RA**	Interleukin-1 receptor antagonist protein	P18510	-0.64	0.62	0.007418	0.33349
**PD1L1**	Programmed cell death 1 ligand 1	Q9NZQ7	-0.65	0.62	0.007569	0.33349
**S10A2**	Protein S100-A2	P29034	-0.66	0.61	0.006288	0.329705
**CYTB**	Cystatin-B	P04080	-0.67	0.61	0.027167	0.47141
**CD9**	CD9 antigen	P21926	-0.68	0.60	0.000227	0.069074
**HMGA1**	High mobility group protein HMG-I/HMG-Y	P17096	-0.68	0.60	0.000725	0.120011
**CADH3**	Cadherin-3	P22223	-0.68	0.60	0.017739	0.442739
**SPIT1**	Kunitz-type protease inhibitor 1	O43278	-0.69	0.60	0.00149	0.159746
**AGRP**	Agouti-related protein	O00253	-0.70	0.59	0.01087	0.37346
**DDB2**	DNA damage-binding protein 2	Q92466	-0.72	0.59	0.014364	0.419263

**ADA15**	Disintegrin and metalloproteinase domain-containing protein 15	Q13444	-0.74	0.58	0.002307	0.221183
**TRML1**	Trem-like transcript 1 protein	Q86YW5	-0.74	0.58	0.005315	0.329705
**NTF4**	Neurotrophin-4	P34130	-0.74	0.58	0.006056	0.329705
**TNR18**	Tumor necrosis factor receptor superfamily member 18	Q9Y5U5	-0.77	0.56	0.020822	0.455168
**TNF6B**	Tumor necrosis factor receptor superfamily member 6B	O95407	-0.77	0.56	0.024232	0.455168
**TNFL6**	Tumor necrosis factor ligand superfamily member 6	P48023	-0.79	0.56	0.005075	0.329705
**TNR18**	Tumor necrosis factor receptor superfamily member 18	Q9Y5U5	-0.80	0.55	0.006792	0.33099
**CLD1**	Claudin-1	O95832	-0.80	0.55	0.016218	0.429842
**KIT**	Mast/stem cell growth factor receptor Kit	P10721	-0.82	0.54	0.007886	0.33349
**VTDB**	Vitamin D-binding protein	P02774	-0.84	0.54	0.034213	0.510999
**MIF**	Macrophage migration inhibitory factor	P14174	-0.94	0.50	0.000902	0.126351
**I18BP**	Interleukin-18-binding protein	O95998	-0.97	0.49	0.000542	0.098827
**CALB1**	Calbindin	P05937	-0.97	0.49	0.023434	0.455168
**LEP**	Leptin	P41159	-1.06	0.46	0.000471	0.095348
**PGH2**	Prostaglandin G/H synthase 2	P35354	-1.34	0.37	0.001115	0.145082
**CEBPA**	CCAAT/enhancer-binding protein alpha	P49715	-1.37	0.36	0.000279	0.07255
**EDN1**	Endothelin-1	P05305	-1.75	0.27	0.003407	0.245555

Abbreviations: FC, fold change calculated from logFC; logFC, logaritmic fold change calculated on the basis of 2; adj.p-val, p-values adjusted for multiple testing

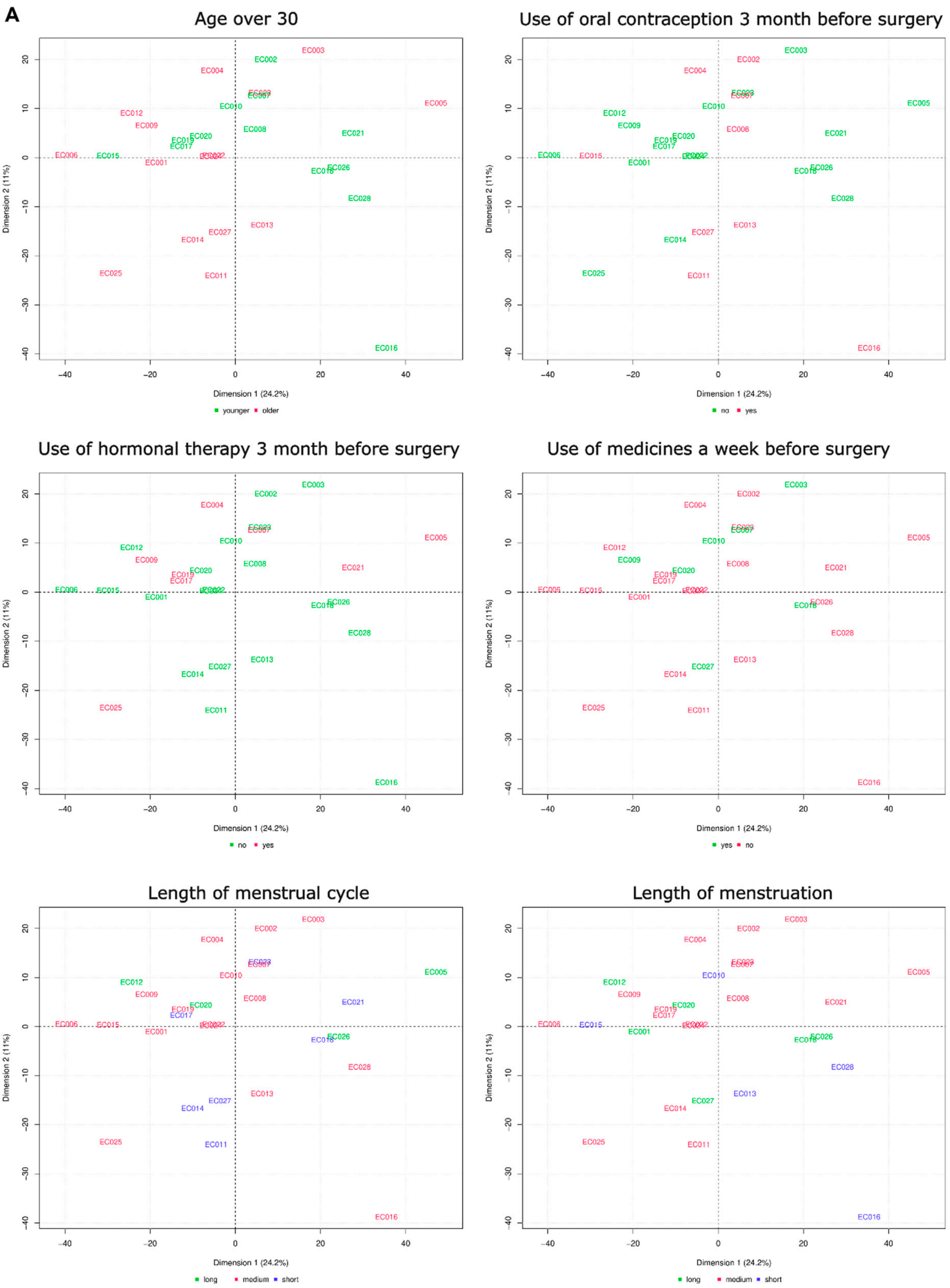
A
Age over 30
Use of oral contraception 3 month before surgery
Use of hormonal therapy 3 month before surgery
Use of medicines a week before surgery
Length of menstrual cycle
Length of menstruation
Dimension 1 (24.2%)
Dimension 2 (11%)
younger older
no yes
no yes
yes no
long medium short
long medium short

B

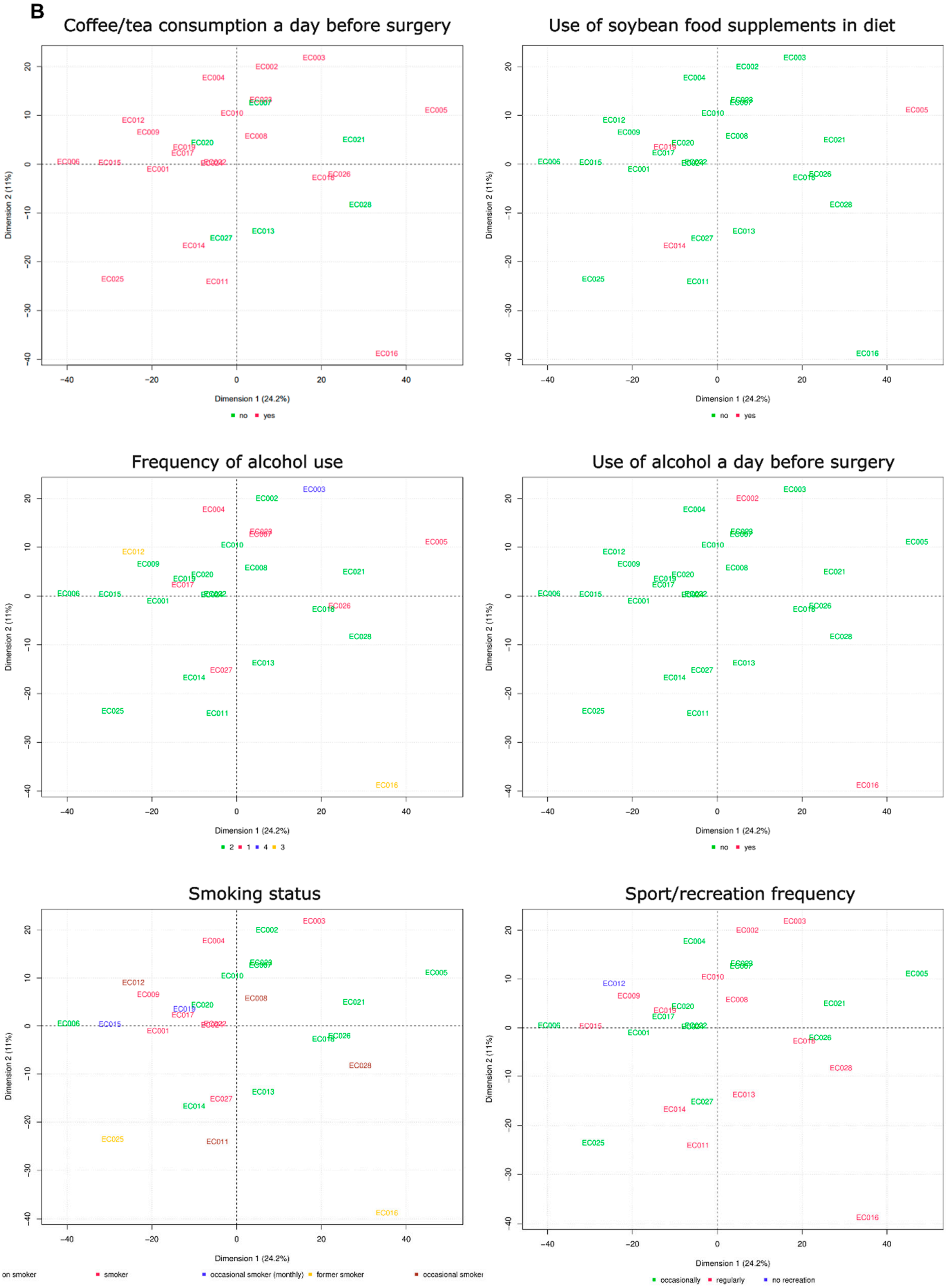

Legend: 1 - Never, 2-Rarely, 3-Once a week, 4- Two to three times a week, 5- More than 3 times a week

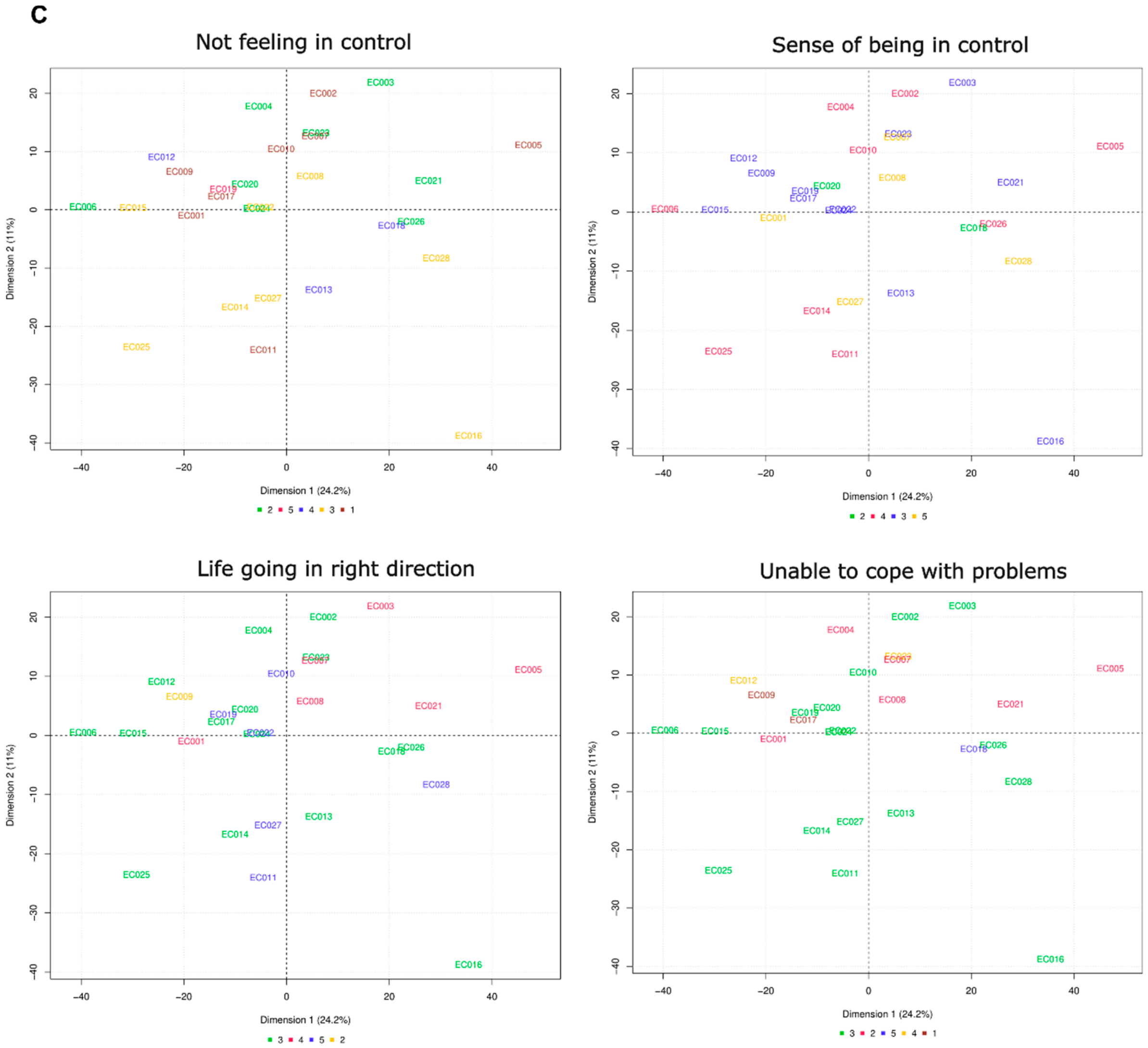

Legend: 1 - Never, 2-Almost never, 3-Sometimes, 4-Quite often, 5- Very often

Figure S1. PCA analysis based on complete array data and different patient characteristics . A) gynecological characteristics B) diet and lifestyle characteristics and C) impact of stress. The presented data are for the secretory group.

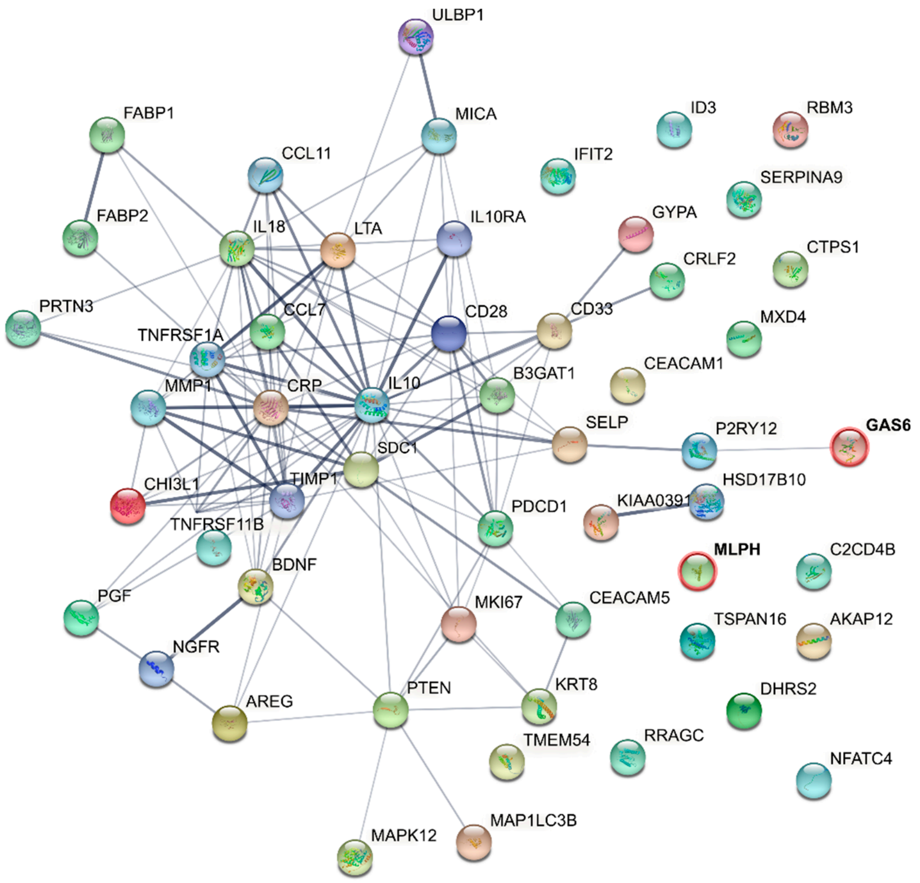

Figure S2. Interactions of the identified differential proteins between patients with peritoneal endometriosis and controls patients in the secretory group. The network includes proteins that reached difference between cases and controls based on log FC > 0.5 and unadjusted $p < 0.05$. The confidence score threshold was set at 0.4 (medium). The figure was acquired online at http://string-db.org/, accessed on January 18 2022 [28].

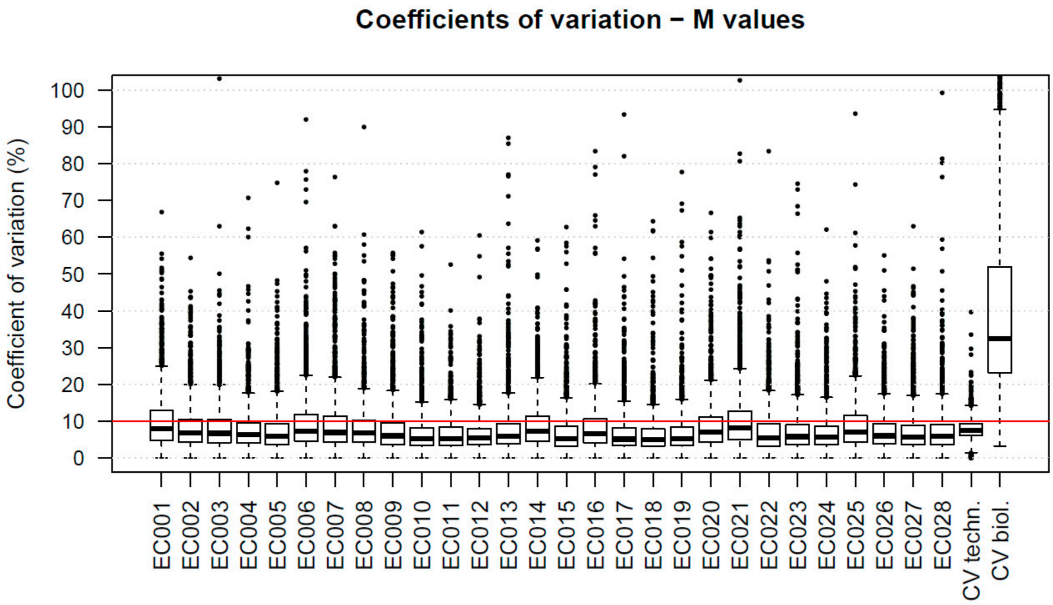

Figure S3. Array-specific distributions of coefficients of variation (CV) for the four technical replicates measured by each antibody. The two rightmost columns represent the average technical variation, i.e. the distribution of the antibody-specific CVs over all arrays (CV techn.), and the biological variation across all samples (CV biol.), respectively.
